# The postnatal leptin surge in mice is variable in both time and intensity and reflects nutritional status

**DOI:** 10.1038/s41366-021-00957-5

**Published:** 2021-09-02

**Authors:** Alicja A. Skowronski, Evan D. Shaulson, Rudolph L. Leibel, Charles A. LeDuc

**Affiliations:** 1 Division of Molecular Genetics, Department of Pediatrics, Vagelos College of Physicians and Surgeons, Columbia University Irving Medical Center, New York, NY USA; 2 Naomi Berrie Diabetes Center, Columbia University Irving Medical Center, New York, NY USA

**Keywords:** Obesity, Homeostasis, Obesity

## Abstract

**Background/objectives:**

The murine postnatal leptin surge occurs within the first 4 weeks of life and is critical for neuronal projection development within hypothalamic feeding circuits. Here we describe the influence of nutritional status on the timing and magnitude of the postnatal leptin surge in mice.

**Methods:**

Plasma leptin concentrations were measured 1–3 times per week for the first 4 weeks of life in C57BL/6J pups reared in litters adjusted to 3 (small), 7–8 (normal), or 11–12 (large) pups per dam fed breeder chow or raised in litters of 7–8 by dams fed high-fat diet (HFD) ad libitum starting either prior to conception or at parturition.

**Results:**

Mice raised in small litters become fatter than pups raised in either normal or large litters. The leptin surge in small litter pups starts earlier, lasts longer, and is dramatically larger in magnitude compared to normal litter pups, even when leptin concentrations are normalized to fat mass. In mice reared in large litters, weight gain is diminished and the surge is both significantly delayed and lower in magnitude compared to control pups. Pups reared by HFD-fed dams (starting preconception or at parturition) are fatter and have augmented leptin surge magnitude compared to pups suckled by chow-fed dams. Surge timing varies depending upon nutritional status of the pup; the source of the surge is primarily subcutaneous adipose tissue. At peak leptin surge, within each group, fat mass and plasma leptin are uncorrelated; in comparison with adults, pups overproduce leptin relative to fat mass. Plasma leptin elevation persists longer than previously described; at postnatal day 27 mice continue overproducing leptin relative to fat mass.

**Conclusions:**

In mice, small litter size and maternal HFD feeding during the perinatal period augment the plasma leptin surge whereas large litter size is associated with a delayed surge of reduced magnitude.

## Introduction

Obesity is a major public health concern with an alarming obesity prevalence of 38% in US adults and 17% in US youth [1]. Obese children and adolescents are five times more likely to become obese adults compared to peers at normal weight [2]. While genetics [3], developmental factors [4], and environmental factors (i.e., energy-dense diet) [5] alone are important contributors to obesity, the interactions among genes, diet, and nutritional and metabolic signals during the perinatal development likely work in synergy to affect body weight later in life.

The hormone leptin is primarily produced and secreted by adipose tissue [6] in proportion to stored fat and its primary function is defense of energy stores and reproductive integrity by signaling peripheral energy availability to the central nervous system (CNS) [7, 8]. In rodents, circulating leptin concentrations between P0 and P22 influence future adiposity: specifically, experimental elevation of circulating leptin concentrations in suckling mice causes mice to gain more weight in adulthood when exposed to high-fat diet (HFD) [4]. During the first 3 weeks of life, in mice, leptin plays a critical role in the generation of hypothalamic feeding circuits by influencing neurogenesis, axon growth, and synaptogenesis [9, 10]; additionally, this postnatal leptin surge is essential for the formation of beige adipocytes during development [11]. Deficiency of leptin during development impairs the formation of projections from the arcuate nucleus of hypothalamus (ARH) to other brain regions involved in energy homeostasis such as the paraventricular nucleus (PVH), the dorsomedial hypothalamic nucleus (DMH), and the lateral hypothalamic area (LHA) [9]. An excess or deficiency of leptin during development alters the formation of projections in these circuits [12–16].

Ahima et al. demonstrated that between postnatal day 7 and 10 mice experience a five- to tenfold surge in circulating leptin concentrations that is unrelated to fat mass or food intake [17]. Early underfeeding (by increasing litter size) and overfeeding (be reducing litter size) of rodents decreases and increases circulating leptin concentrations, respectively, during the first 3 weeks of life [18–20]. In rodents, maternal HFD feeding during gestation and lactation augments the postnatal leptin surge in the progeny [13, 21, 22]; and maternal caloric restriction reduces body weight of pups and reduces their postnatal leptin surge [14]. In rodents, maternal HFD feeding is associated with disruption of the normal pattern of projections in the hypothalamic feeding circuits, including decreased AgRP immunoreactive fibers in the PVH [12, 13], and reduced density of α-MSH projections from the ARH to PVH, DMH and LHA in 8-week-old progeny [12]. These same projections are disrupted in congenitally leptin deficient mice, suggesting that the effects of nutritional state during the postnatal period on adult body weight may be mediated through effects on the postnatal leptin surge.

We hypothesized that the nutritional status of the postnatal pups affects the timing and magnitude of the leptin surge. We determined how the leptin surge is affected by early over- or undernutrition and maternal HFD feeding during the first 4 weeks of life and how it is affected by fat mass of the pup. We conclude that postnatal overfeeding and maternal HFD feeding augment the leptin surge whereas postnatal underfeeding diminishes and delays the surge even when adjusted for the fat mass of pups.

## Methods

### Animals

Experiments were conducted on C57BL/6J mice (Stock no: 000664 | B6) purchased from Jax. Throughout the study, animals were maintained at room ambient 22–24 °C with a 12-h dark–light cycle (lights on at 0700 h) in a pathogen-free barrier facility. Mice were fed breeder chow (LabDiet 5053) ad libitum throughout the experiment unless a different diet is specified. All pups were weaned on postnatal day P22. The protocol was approved by the Columbia University Institutional Animal Care and Use Committee.

### General study design

Pregnancies were timed so that all pups were born on the same day of the week. On P2, pups were redistributed across the lactating dams (to minimize gestational environmental bias) in specified litter sizes. For the purposes of this manuscript, “normal” sized litter is considered 7–8 [23] pups per nursing mother, while the “large” and “small” litters were adjusted to 11–12 or 2–3 nursing pups per mother, respectively. On P3–P4, pups were tattooed on the paws and a subset were weighed for the first time. Starting on P6, pups were weighed and body composition was measured three times per week. A subset of pups (primary cohort) had blood collected three times a week for the first month of life. Other pups were bled once a week (replication cohort and maternal HFD feeding cohort) so that each litter had pups bled on all 3 weekly time points to minimize weight perturbations in the pups due to handling for blood collection. On P22 pups were separated by sex and weaned to new home cages with ad libitum chow and water access. Body weight and composition in the weaned mice were monitored for an additional week. Due to the nature of experiments reported in this paper, it was not feasible for the investigators to be blinded to the group allocation.

### Effects of over- and undernutrition on leptin surge

**Fig. 1:**
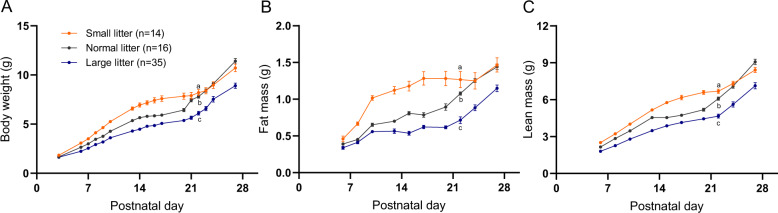
Growth profiles of C57BL/6J mouse pups during the postnatal period. Body weight (**A**), body fat (**B**), and lean mass (**C**) in the primary cohort of mixed sexes pups raised in small (SL; three pups per dam; orange), normal (NL; 7–8 pups per dam; gray), and large (LL; 11–12 pups per dam; blue) litters monitored over the first 4 weeks of life and bled 3 times per week. GEE analysis performed to compare changes in body weight and composition over the pre-weaning period while pups were in their respective litter size groups; statistics in supplementary Table 2. Different lowercase letters indicate significant difference by the Wald Chi-Square test at the 5% level of significance. Data shown as mean ± SEM.

C57BL/6J mice purchased from Jax were bred while maintained on breeder (13% of calories from fat) chow. As per protocol above, after pups were born, litter size was adjusted to generate small (SL), normal (NL) and large litters (LL). We did not detect any significant differences in the plasma leptin concentration (Fig. S1), body weight or body composition between male and female pups. Therefore, the data were analyzed with pooled sexes. In the primary cohort, mice were bled 3 times a week to provide fine temporal resolution of leptin concentrations in individual mice. In the replication cohort, the mice were bled once a week on a rotating schedule to match the same time points, but each mouse was only bled once per week. While we noted that the body weight and composition in SL were affected by 3 times per week blood sampling, the plasma leptin concentrations were similar between the primary and replication cohorts (Figs. 1, S2). Plasma insulin and glucose concentrations were measured on P10 in the primary cohort. Due to limited amounts of plasma, insulin (*n* = 15–20 per group) and glucose (*n* = 9–11 per group) were measured in a subset of pups. The following are the number of pups constituting each cohort: Primary: (1) SL: 14 pups, (2) NL: 16 pups, and (3) LL: 35 pups. Replication: (1) SL: 19 pups, (2) NL: 45 pups, and (3) LL: 22 pups. Sample size was based on similar studies carried out in our laboratory. Data for all animals used were included in the analyses except animals that did not survive the postnatal period. In total seven pups died: three in SL, three in NL, and one in LL.

A separate cohort of SL, NL, and LL mice was created and the pups were sacrificed at P10 for gene expression analysis in the following adipose tissue depots: subcutaneous (SCAT), perigonadal (PGAT), mesenteric (MES), perirenal (PR), interscapular brown (iBAT), and axillary brown (aBAT). Pups were weighed, bled, analyzed by MRI for body composition, and then sacrificed by cervical dislocation; SCAT and iBAT depots were weighed for each pup (the other depots were <5% the mass of the SCAT) and all AT depots were flash frozen for RNA extraction.

### Effects of maternal high-fat diet on leptin surge

Leptin surge was measured in pups born to: (1) dams maintained on breeder chow and switched to HFD (Research Diets, Inc. D12492i; 60% calories from fat; HFD) at parturition (HFD at parturition group), and (2) dams fed HFD ad libitum starting at 6 weeks of age for ~20 weeks through gestation and lactation (HFD preconception group). All pups were reared in litters of 7–8 pups per dam (normal size litter). The experimental design for this set of mice was the same as for the small/normal/large size litters bled once a week, therefore, a subset of pups reared in NL by dams maintained on breeder chow was used as a control group (chow control) for the pups reared by HFD-fed dams. Body weight, composition and plasma leptin concentrations (Fig. S3) were not significantly different; therefore, all data is presented with mixed sexes. The following number of pups (mixed males and females) were included in the study for each group: (1) chow control: 30 pups, (2) HFD at parturition: 35 pups, and (3) HFD preconception: 43 pups. Data for all animals used were included in the analyses except animals that did not survive the postnatal period. In total four pups died: one in HFD at parturition and three in HFD preconception groups.

### Body composition

Body weight and composition were measured using a laboratory scale (accurate to 0.01 g) and an EchoMRI Body Composition Analyzer, respectively, immediately before blood collection. Body composition for large litter in the replication cohort for the various size litter experiment was only collected up to P13 due to technical issues with the EchoMRI.

### Plasma collection and assays

Approximately 20 µl of whole blood was obtained by submandibular bleed at 9–10 a.m. from mice in a fed state. Blood was collected on ice using heparinized tubes (Fisherbrand) and plasma was isolated by centrifugation for 20 min at 2000 × *g* at 4 °C, aliquoted, and frozen at −80 °C until time of assay. Plasma leptin concentration was measured using mouse leptin ELISA (R&D) with sensitivity of 22 pg/ml. Plasma insulin concentration was measured using mouse insulin ELISA (Mercodia) and plasma glucose was measured with Autokit Glucose (WAKO).

### RNA extraction and gene expression

AT samples were homogenized in TRIzol™ Reagent (#15596026), mixed with chloroform and centrifuged for phase separation. RNA-containing aqueous phase was mixed with equal volume of 100% ethanol and RNA was isolated using the RNA Clean and Concentrator-5 kit (Zymo Research #R1016). RNA was reverse-transcribed with Transcriptor First Strand cDNA kit (Roche # 04897030001) and cDNA was diluted 1:10 for qPCR assay. Standard curves were generated by pooling equal volumes of cDNA from all samples followed by serial dilution. Gene expression quantification was done with PrimeTime qPCR Probe Assays (IDT) using the Roche LightCycler 480 for the following genes: *Lep*, *Lepr*, *Adrb3*, *Lipe*, *Ucp1*, and *Adipoq* (primer and probe sequences provided in Table S1). *Actb* was used as a house-keeping gene and was assayed along with two genes of interest (for a total of three genes per reaction mix).

### Statistical analysis

All data are presented as means with standard error. Data were analyzed using SPSS or GraphPad PRISM. The primary outcome in our experiments is the leptin concentration during postnatal period.

Leptin area under the curve (AUC) for the primary cohort for which blood was sampled 3 times a week was calculated from P6 to P17 for each mouse, while for the replication cohort (once a week bleeding), leptin AUC was calculated on a mean per group basis over the same time period. For the maternal HFD feeding experiment in which any given mouse was bleed only once a week the leptin AUC was calculated over a 2-week period but the exact timing for any given mouse depended on the time of bleeding; therefore, leptin AUC for each mouse was calculated for one of 3 time periods: P6–P20, P8–P22, or P10–P24. Leptin AUC was then normalized to mean fat mass over the same period. Leptin peak was determined for each mouse based on the maximum point measured over the first 4 weeks of life. Leptin peak was then adjusted for by body weight or fat mass and by the age at which the peak occurred.

Leptin AUC, leptin peak, insulin and glucose were analyzed using General Linear Model/Univariate in SPSS software which is a Univariate Analysis of Variance (with covariates when appropriate) followed by either Tukey HSD (for ANOVA) or Sidak (for ANOVA with covariates) post hoc analysis to correct for multiple comparisons. *P* < 0.05 was considered significant.

To account for correlation between the repeated measures, longitudinal continuous data (body weight and body composition) was analyzed using the Generalized Estimating Equations (GEE) with exchangeable correlation structure. Time was included as a covariate. Student’s t-test was used to compare body weight and body composition between the groups at specific time points determined a priori (at P10 and P22, the approximate time of leptin peak and at weaning, respectively).

As mentioned in “Body Composition” section, LL in the replication cohort had MRI data collected until P13, therefore, the GEE analysis between LL, NL and SL was carried out from P6 to P13 to compare LL vs. NL and LL vs. SL but a separate GEE analysis was performed to compare NL and SL from P6 to P27 (since only these groups had all datapoints available).

Body weight, body composition, and leptin concentrations were compared between males and females and no significant differences were detected in any of these parameters therefore the data is presented and analyzed with mixed sexes in each dietary group.

## Results

### Effects of over- and undernutrition on leptin surge

In the first 3 weeks of life, prior to the leptin surge, mice do not regulate food intake [24]; they ingest all the milk that is available. In SL (*n* = 2–3 pups per nursing mother), pups have greater access to milk and gain more weight than LL (*n* = 11–12 pups), or NL (*n* = 7–8 pups per nursing mother) during the pre-weaning period (Figs. 1A, S2a) [25]. Differences in body weight are accounted for by both fat and lean components of body mass (Figs. 1B, C, S2b, c). Prior to weaning, body weight, fat mass and lean mass increments over time were significantly different among the three groups (GEE results Table S2).

**Fig. 2:**
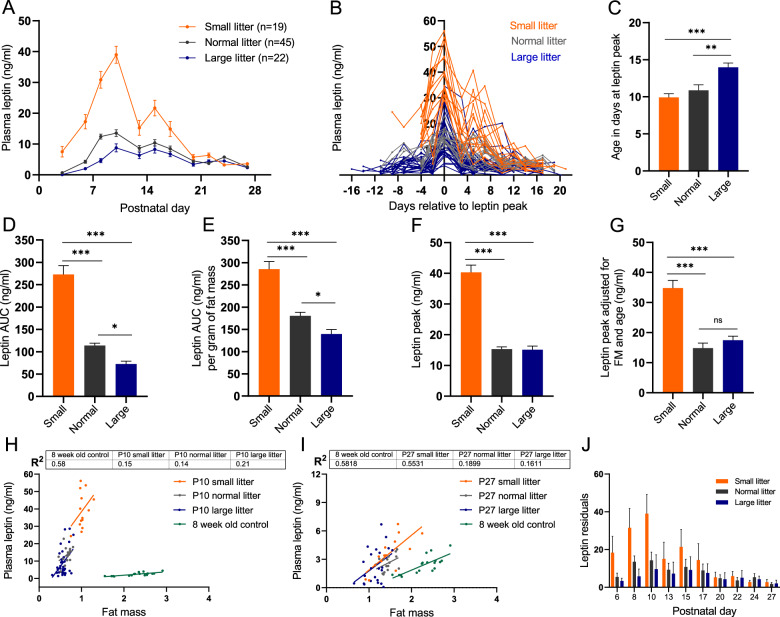
Over- and undernutrition effects on leptin surge in the primary cohort bled 3 times per week. **A** Mean plasma leptin, (**B**) plasma leptin in individual pups with leptin peak for each mouse plotted at *x* = 0, (**C**) mean age of pups at which the leptin peak occurred, (**D**) mean leptin AUC from P6 to P17, (**E**) mean leptin AUC from P6 to P17 normalized to animal’s mean fat mass over that time period, (**F**) mean leptin peak defined as the maximum leptin concentration measured during the first 4 weeks of postnatal life, (**G**) mean leptin peak adjusted for fat mass and age at which the peak occurred in mix sexes pups raised in small (SL; three pups per dam; orange), normal (NL; 7–8 pups per dam; gray), and large (LL; 11–12 pups per dam; blue) litters monitored over the first 4 weeks of life. Regression of fat mass vs. plasma leptin in P10 (**H**) and P27 (**I**) pups from SL, NL, or LL, as well as 8-week-old adult control mice fed regular chow and raised in normal litters for comparison. **J** Leptin residuals for SL, NL, and LL pups calculated based on leptin predicted from the fat mass vs. leptin of 8-week-old control adult mice fed regular chow diet and raised in normal litters, and observed plasma leptin concentration for SL, NL, and LL at each time point. Difference in leptin AUC and leptin peak among the groups tested using one-way ANOVA with Tukey post hoc (or Sidak for ANOVA with covariates) at the 5% level of significance; *p < 0.05; **p < 0.01, ***p < 0.001. Means shown with ±SEM.

The magnitude of the leptin surge in SL pups was greater than in NL and the NL surge was greater than in LL (Fig. 2). Leptin AUC from P6 to P17 (when the leptin surge is most pronounced), was 2.4- and 3.8-fold higher in the SL than in the NL and LL pups, respectively (Fig. 2D). SL pups mean fat mass was 1.5-fold higher during the leptin surge period compared to NL pups. Leptin AUC normalized to mean fat mass was still 1.6-fold higher in SL than in NL; the plasma leptin elevation in SL was higher than predicted from the increase in fat mass (Fig. 2E). The magnitude of the absolute leptin peak in SL was 2.7-fold higher than in NL and LL, and this difference persisted even when leptin peak was adjusted for fat mass and age at which the peak occurred (Fig. 2F, G). In SL and NL, maximum plasma leptin concentration occurred around P10; in SL pups circulating leptin concentrations reached those of the NL peak as early as P6 (leptin in SL at P6 is 17.3 ng/ml compared to 15.3 ng/ml in NL at leptin peak) indicating that the leptin surge began much earlier in SL pups (Fig. 2A–C).

Leptin AUC in LL pups was 40% lower compared to NL and remained 20% lower when normalized to fat mass. Additionally, while the magnitude of leptin peak in LL was the same as in NL, its timing was delayed by 3 days (Fig. 2). These data suggest that underfeeding (LL) does not reduce the magnitude of the peak leptin surge, but that the overall amount of leptin (AUC) to which the pups are exposed during the postnatal period is lower compared to NL pups; and that the onset of the peak occurs significantly later (Fig. 2).

In SL pups, the leptin surge (mean leptin concentration at each time point) has a sharp peak at P10 (Fig. 2A). In contradistinction, NL and LL display broader elevations of plasma leptin extending over the 2nd and 3rd postnatal week. Important information regarding the leptin surge in individual mice is lost when circulating leptin is averaged per group. Bleeding every mouse three times a week in the primary cohort provided detailed temporal resolution of circulating leptin on an individual basis (Fig. 2B). Figure 2B synchronizes the leptin surge; the peak leptin day for each individual mouse was plotted as *x* = 0; this figure indicates that there is a discrete leptin peak for mice in all groups. Figure 2B also demonstrates that the leptin peak occurs on different postnatal days (especially in NL and LL) with variable magnitude for each mouse; the predominant pattern differs among the groups. In SL, the leptin surge begins earlier. The majority of SL pups peak on P10 (Fig. 2B) and this peak is higher (the peak circulating leptin ranges from 29 to 56 ng/ml compared to the NL range of 11 to 23 ng/ml), and lasts longer compared to NL (Fig. 2B). In LL pups, the magnitude of the peak leptin surge varies widely among the pups (4–35 ng/ml) and, on average, occurs later (Fig. 2B, C, F, G). In LL mice, the shorter duration of the leptin peak may have obscured the detection of the absolute peak, resulting in an apparently lower observed leptin maxima in some LL mice.

In the replication cohort, plasma leptin was measured once per week in each pup to reduce the stress of frequent blood sampling. Body weights in SL and LL were significantly higher and lower, respectively, compared to the NL throughout the first month of life (Fig. S2a, GEE results in Table S3). Body fat and lean mass were significantly higher in SL compared to NL throughout the study (GEE results in Table S3, Fig. S2b, c). Body composition data were collected up to P13 in LL. Lean and fat mass in LL were significantly lower over that period (P6 to P13) compared to NL and SL (GEE results in Table S3). The effect of litter size on body weight and composition, especially in the SL, was greater in the replication vs. the primary cohort and was directly related to the frequency of blood sampling.

In the replication cohort the leptin peaks and AUCs were significantly higher in SL compared to NL and LL (Fig. S4b–e). Because we did not measure fat mass in all postnatal time points in the LL (see Methods), we normalized leptin AUC to body weight as a proxy for fat mass and—consistent with the primary cohort—found this parameter to be significantly higher in SL compared to NL and LL. In the replication cohort the number of blood samplings per time point was decreased by a factor of 3. With the smaller sample sizes, we did not detect a statistically significant difference in leptin AUC (normalized for body weight) in LL vs. NL. Similar to the primary cohort’s results, we did not detect a significant difference in leptin peak (either absolute or adjusted for body weight and age) between LL and NL pups (Fig. S4d, e). While sampling blood 3 times per week, as described in the primary cohort, affected body weight and composition in SL, plasma leptin concentrations at the respective time points were not different between the primary and replication cohorts (Figs. 2, S4).

The increase in postnatal plasma leptin occurs coincidentally with the formation of hypothalamic leptin response circuitry. In adult mice, circulating leptin is proportional to white adipose tissue (WAT) mass; however, the amount of leptin produced by pups at the peak of the leptin surge (in all 3 groups) is much higher than would be predicted from their WAT mass assuming proportionality to the adult (Fig. 2H). Also, at the peak of the leptin surge (~P10), within each litter size group, fat mass and circulating leptin concentrations are essentially uncorrelated (Fig. 2H). Despite the 8-week-old adult mice having a 3.5-fold higher fat mass on average compared to P10 NL pups, circulating leptin concentrations are substantially lower with almost no overlap in leptin concentrations between the two groups (Fig. 2H). Using a regression equation relating plasma leptin to fat mass in the 8-week-old adult mice, we estimated the anticipated leptin concentration for each nursing pup on each postnatal day based on their fat mass. Between P6 and P27, the actual circulating leptin was higher than predicted from the adult regression equation in every litter size group. On P27, the residual leptin concentrations (measured leptin minus expected from regression based on 8-week-old mice) in NL were greatly reduced from the leptin surge peak, but were still 1.8 ng/ml higher than predicted from the adult regression equation (Fig. 2I, J). These data suggest that the postnatal leptin surge has a longer duration than was previously reported.

**Fig. 3:**
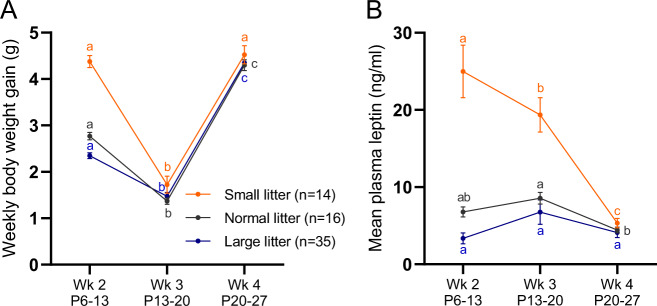
Weekly weight gain and plasma leptin during postnatal period. Body weight gain (**A**) and mean plasma leptin concentration (**B**) in SL, NL, and LL pups during second (P6–13), third (P13–P20), and fourth (P20–P27) week of life from the pups bled once per week. Means marked by a common letter are not significantly different by two-way ANOVA followed by Tukey post hoc test at the 5% level of significance. The *post hoc* comparisons are made only between different weeks within each litter group. Data shown as mean ± SEM.

In all litter size groups, the weekly rate of weight gain was not consistent throughout the first 4 weeks of life. In the first 2 weeks of life this weight gain was not correlated with absolute circulating leptin concentrations (Fig. 3A, B). While pups gained 2.5-, 2-, and 1.6-fold more weight in the 2nd week compared to the 3rd week in SL, NL, and LL, respectively, the mean concentrations of leptin during the 2nd and 3rd week were high (relative to both their circulating leptin concentrations in the 1st and 4th week and their anticipated plasma leptin predicted from fat mass) during both weeks and were not significantly different for NL or LL mice. In SL mice circulating leptin concentrations were significantly lower in the 3rd week vs. 2nd week, but in the 3rd week were still substantially elevated compared to week 4 or to any other time point in the NL or LL groups (Fig. 3). Bouret et al. showed that the leptin-dependent neuronal projections from the ARH to DMH, PVH, and LHA are apparent by the 2nd week of life [9]. Our data indicate that after the peak of circulating leptin in the 2nd postnatal week, mice gain weight more slowly during the 3rd postnatal week; this change could be an indication of mice beginning to respond to leptin by inhibiting food intake (Fig. 3). The velocity of weight gain increases in the 4th week, and is 2.6-, 3.1-, and 3-fold higher in SL, NL, and LL, respectively, than in the previous week; this acceleration of weight gain coincides with a decrease in circulating leptin concentration, supporting the idea that the ARH neuronal projections forming during the 2nd week in response to surging leptin concentrations may provide the neural substrate for the subsequent role of leptin in the regulation of food intake.

**Fig. 4:**
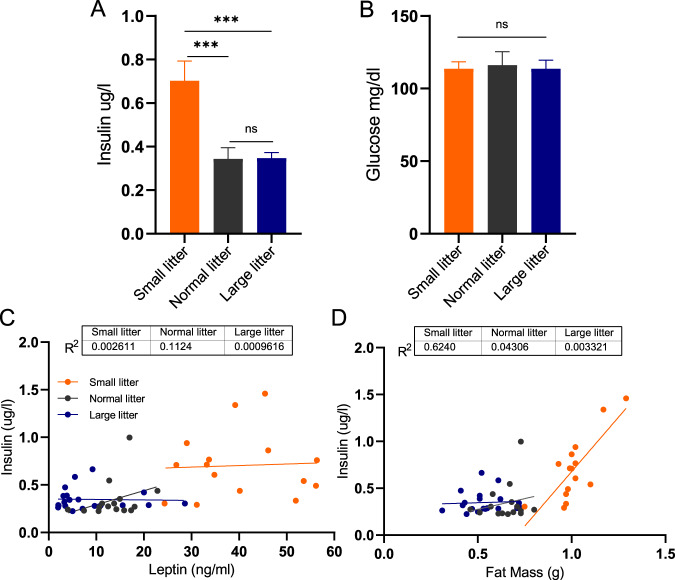
Over- and undernutrition effects on circulating insulin and glucose concentrations at P10. Mean plasma insulin (*n* = 15–20 mice per group) (**A**) and venous plasma glucose (*n* = 9–11 mice per group) (**B**) in pups raised in small (orange), normal (gray), or large (blue) litters measured on postnatal day 10 during the peak of plasma leptin surge. Regression of plasma leptin concentrations (**C**) or fat mass (**D**) vs. insulin for each of the litter size group. Difference in plasma insulin and glucose concentrations among the groups were tested with one-way ANOVA with Tukey post hoc at the 5% level of significance; ***p < 0.001; ns denotes nonsignificant difference.

We investigated the relationship of the leptin surge to plasma insulin and glucose concentrations at P10, the height of leptin surge. While plasma insulin was twice as high in SL as in both NL and LL, venous plasma glucose concentrations were not different among the three groups (Fig. 4A, B). Plasma insulin concentrations did not correlate with circulating leptin concentrations in any of the groups. Interestingly, in SL animals, circulating insulin concentrations were more highly correlated with fat mass (*R*^2^ = 0.6) than with leptin concentrations (*R*^2^ = 0.003; Fig. 4C, D).

**Fig. 5:**
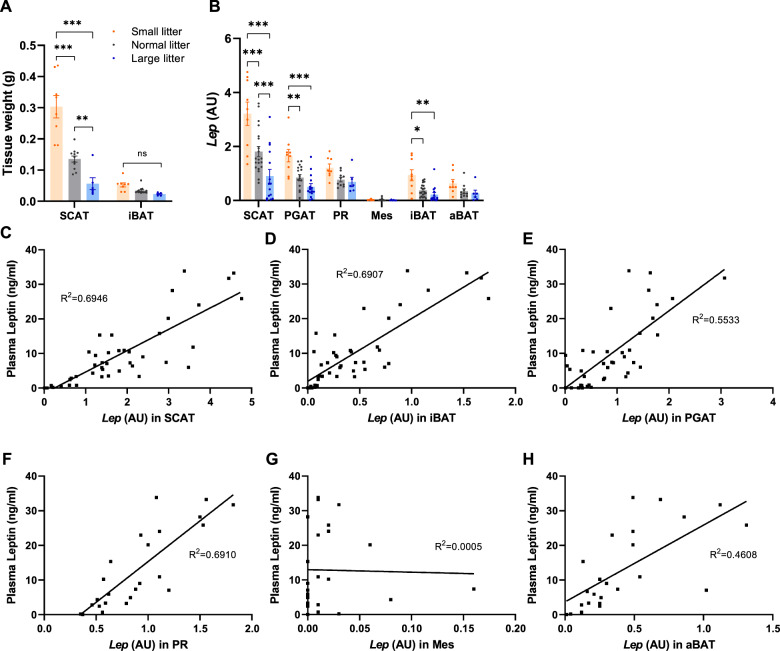
*Lep* expression in adipose tissue depots and its correlation with circulating plasma leptin in small, normal, and large litter pups. Weight of SCAT and iBAT tissue (**A**) and *Lep* expression (**B**) in various adipose depots in P10 pups reared in small, normal or large litters. Correlation of *Lep* expression vs. plasma leptin in SCAT (**C**), PGAT (**D**), PR (**E**), Mes (**F**), iBAT (**G**), and aBAT (**H**) in P10 pups (small, normal or large litters). Differences among the groups were tested with one- or two-way ANOVA with Tukey post hoc testing at the 5% level of significance; *p < 0.05; **p < 0.01; ***p < 0.001, ns denotes nonsignificant difference. SCAT subcutaneous adipose tissue, PGAT perigonadal adipose tissue, PR perirenal adipose tissue, Mes mesenteric adipose tissue, iBAT interscapular brown adipose tissue, aBAT axillary brown adipose tissue.

We created an additional cohort of pups reared in SL, NL, or LL and sacrificed at P10 (the mean day at which leptin peak occurs in pups from normal size litters). The mass of subcutaneous WAT (SCAT) depot was significantly greater in SL and less in LL vs. SCAT in NL pups (Fig. 5A). Interscapular BAT (iBAT) weight was not significantly different among the groups (Fig. 5A). The weights of perigonadal (PGAT), perirenal (PR), mesenteric (Mes), and axillary (aBAT) fat depots were not recorded but combined to a mass less than 5% of the mass of the SCAT. Pups from SL had significantly higher expression of *Lep* in SCAT, PGAT and iBAT compared to NL, while in LL, expression was significantly lower only in SCAT vs. NL (Fig. 5B). No significant differences in *Lep* expression—by litter size—were detected in PR, Mes and aBAT depots.

Circulating leptin concentration correlated with *Lep* expression in all adipose tissues except for Mes (SCAT, *R*^2^ = 0.695; iBAT, *R*^2^ = 0.691, PGAT, *R*^2^ = 0.553; PR, *R*^2^ = 0.691; Mes, *R*^2^ = 0.0005; aBAT, *R*^2^ = 0.461; Fig. 5C–H). SCAT is by far the largest AT depot in P10 pups (Fig. 5A) and expresses *Lep* on a nominal per cell basis at the highest level compared to other AT (Fig. 5B). Together, these data suggest that SCAT is the largest contributor to the leptin surge in postnatal pups.

We also measured expression of *Lepr*, *Adrb3*, *Ucp1*, *Hsl*, and *Adipoq* in all adipose depots (Fig. S5). *Adrb3* was significantly lower in SCAT of SL pups compared to NL and LL, consistent with presumed decreased metabolic demand for lipolysis (Fig. S5). *Ucp1* was significantly higher in BAT of SL vs. NL and LL pups. In large litter animals, *Hsl* was expressed at a higher level in PR, iBAT and aBAT. *Adipoq* expression in SCAT and iBAT showed an inverse relationship to adiposity of the pups, consistent with the pattern reported in adult animals (Fig. S5) [26, 27].

### Effects of maternal high-fat diet feeding on time and magnitude of leptin surge

**Fig. 6:**
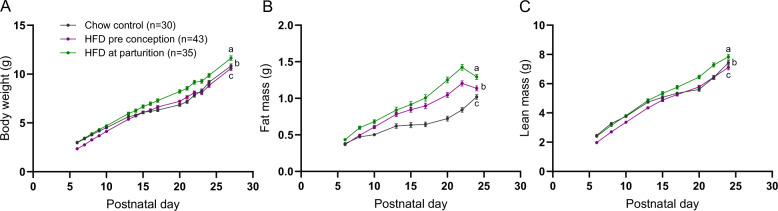
Growth profiles of C57BL/6J pups reared by chow- or HFD-fed dams during the postnatal period. Body weight (**A**), body fat (**B**), and lean mass (**C**) in mixed sexes pups raised by dams fed HFD either from parturition (green) or for 20 weeks preconception (purple), monitored over the first 4 weeks of life and bled once per week. GEE analysis performed to compare body weight and composition changes over the first 4 weeks of life; statistics in Supplementary Table 4. Different lowercase letters indicate significant difference by the Wald Chi-Square test at the 5% level of significance. Data shown as mean ± SEM.

When dams were maintained on breeder chow throughout life and then switched to HFD at parturition, their pups (“HFD at parturition group”) had significantly larger fat and lean mass compartments compared to pups reared in NL-sized litters by dams fed breeder chow (“chow control group”; GEE results Table S4; Fig. 6). At weaning (P22), body weight, fat mass and lean mass of the offspring were 17%, 69%, and 13% higher in HFD at parturition compared to chow control (all with 7–8 mice per litter) group (Fig. 5). When dams were fed HFD for 20 weeks prior to conception and throughout gestation and lactation, the body weights and lean masses of pups (“HFD preconception” group) were significantly different from chow control pups. The progeny of dams continuously fed HFD had lower body weights during the first 2 weeks of life, but showed greater rates of weight gain than the chow controls thereafter (GEE Results Table S4; Fig. 6A). By weaning at P22, despite no significant difference in body weight and lean mass between the HFD preconception and chow control groups, fat mass at P22 was 44% higher in HFD preconception animals (Fig. 6). In both HFD at parturition and HFD preconception, fat mass of the offspring was decreased at P24 due to a switch in diet from HFD to breeder chow at weaning, P22 (Fig. 6B).

**Fig. 7:**
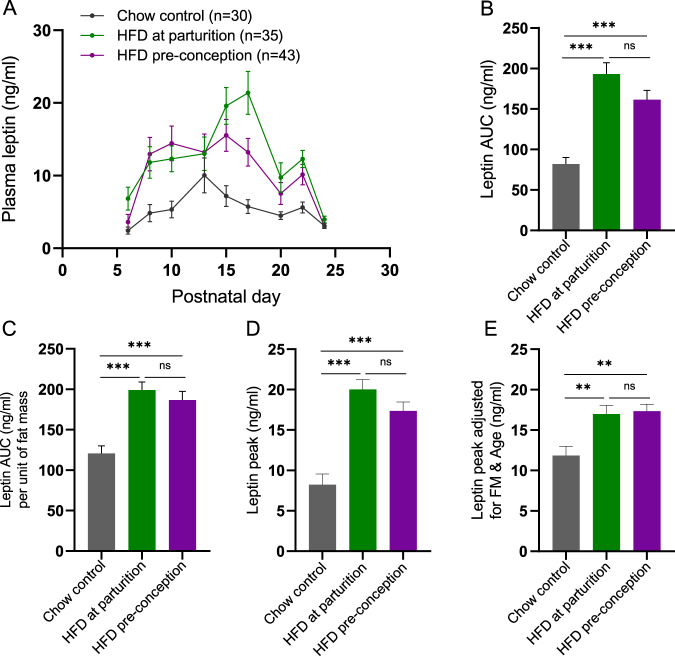
Maternal high-fat diet effects on leptin surge in pups. **A** Mean plasma leptin concentration, (**B**) mean plasma leptin AUC over a 2-week period, (**C**) mean plasma leptin AUC normalized to mean fat mass, (**D**) mean peak leptin concentration (peak defined as maximum leptin concentration during the first 4 weeks of postnatal life), (**E**) mean peak leptin concentration adjusted for fat mass, and age at which the peak occurred in mixed sexes pups raised by dams fed HFD either from parturition (green) or for 20 weeks preconception (purple). Differences in leptin concentration AUCs and leptin peak concentrations among the groups were tested using one-way ANOVA with Tukey post hoc (or Sidak for ANOVA with covariates) at the 5% level of significance; **p < 0.01; ***p < 0.001. Means shown with ±SEM.

In the progeny of dams fed HFD either at parturition or prior to conception through weaning, the leptin AUC was increased 2.4- and 2-fold, respectively, compared to the NL offspring raised by dams maintained on breeder chow (Fig. 7A, B). Leptin AUC in progeny of dams fed HFD at parturition and HFD preconception was 1.6- and 1.5-fold higher, respectively, than in control pups even after normalizing leptin for fat mass (Fig. 7C). Additionally, the leptin peaks were 2.4 and 2.1-fold higher in HFD at parturition and HFD preconception offspring, respectively, compared to the progeny of maternal chow controls (Fig. 7D). After adjusting for the age and fat mass at which the peak leptin concentrations occurred, the significant differences in mean leptin peak height persisted: 1.4- and 1.5-fold higher in HFD at parturition and HFD preconception groups, respectively, vs. chow control group (Fig. 7E).

## Discussion

Our data are consistent with previous reports that the postnatal leptin surge in mice peaks around P10 [17, 18, 21, 28, 29]. Here we demonstrate that the timing, magnitude, and duration of this surge are strongly influenced by the nutritional status of the animals. In C57BL/6J mice raised in conventional litters of 7–8 animals, plasma leptin peaks at P10 and remains modestly elevated (relative to fat mass) until P27. To investigate the effects of nutritional status on the leptin surge we both manipulated litter size and fed dams a HFD either during lactation only, or from 20 weeks prior to conception and continuing through the period of lactation. Others have previously shown that reduced litter size or maternal HFD feeding results in increased body weight and adiposity persisting into adulthood [30–33] and increased weight gain when exposed to HFD [30]. Increasing the number of pups per dam results in decreased weight gain during nursing, and lower body weight as adults [34]. Alterations in body mass during the suckling period can have lifelong consequences for body weight and adiposity. Here, we show that these nutritional perturbations affect the leptin surge and are consistent with previous reports [18–20, 35]. Alterations in the leptin surge associated with SL and LL are correlated with differences in adiposity as adults. The magnitude of leptin surge in small litters (SL) is dramatically higher than in NL, even when normalized to fat mass; a substantial increase in plasma leptin is apparent as early as P3, only 24 h after reducing the litter size. Similarly, maternal HFD feeding, regardless of the timing of onset of HFD exposure (at parturition vs. preconception), is associated with increased magnitude of the leptin surge compared to dams maintained on breeder chow. These data are consistent with prior reports that maternal HFD feeding in rats leads to persistent increases in body weight and an augmented leptin surge [13, 21, 22]. In contrast, pups reared in LL—with limited access to breast milk—display a delayed leptin surge of reduced magnitude. These data indicate that the leptin surge is highly dependent on the nutritional state of the pups but not on their fat mass per se. In the maternal HFD feeding experiment, it is important to note that the control dams were fed breeder chow which contains 24.6% of calories as protein compared to 20% in HFD; it is possible that some of the differences in leptin surge among the pups were mediated by disparate protein content in the diet [36, 37].

Our data are consistent with studies reporting that by the end of the 3rd postnatal week, hypothalamic feeding circuits are anatomically apparent and functionally active, due, at least in part, to the neurotrophic actions of leptin [9]. The number of leptin-sensitive neurons in the ARH increases over the first 3 weeks of life and reach adult levels by P25. Leptin induces PSTAT3 activity in the ARH by P10 [38]. Additionally, electrophysiological brain recordings demonstrate that the NPY/AgRP/Gaba-expressing ARH neurons that are activated by leptin, in the 2nd postnatal week switch to being inhibited by leptin by P30 [38]. Mistry et al. showed that a single intracerebroventricular (ICV) injection of leptin does not inhibit food intake or reduce body weight of P17 pups, but an anorectic response to leptin occurred when pups were ICV injected with leptin at P28 [24]. The body weight of congenitally leptin deficient, Lep^*ob/ob*^, mice is indistinguishable from that of wild-type mice before ~2 weeks of age [39]. In female mice in which leptin concentration is controlled by a TET-On system responding to doxycycline, inducing hyperleptinemia in the first 3 weeks of life does not affect fat mass at P9; but by P16, the fat mass in postnatally hyperleptinemic mice is significantly reduced [4]. In the present study, the relationship of velocity of weight gain to mean weekly circulating leptin provides additional evidence that leptin does not provide an anorectic signal during the first 2 weeks of the suckling period. Our data suggest that between P13 and P20 suckling mice begin to show suppression of weight gain in response to leptin. Leptin-induced maturation of hypothalamic feeding circuits and changes in circulating concentrations resulting from the “surge” and its resolution could account for deceleration in the rate of weight gain during the 3rd postnatal week, and its acceleration in the 4th postnatal week [40, 41].

The physiological basis for the leptin surge is unclear. In adult rodents, leptin is primarily expressed in WAT and to a lesser degree in BAT [42, 43]. At day P10, SCAT is the largest depot by weight in all pups, regardless of litter size. Additionally, on a per cell basis (normalized by *Actb* expression), leptin expression is highest in SCAT compared to all other depots measured (PGAT, PR, Mes, iBAT, and aBAT) at P10. Together these results indicate that the primary driver of the leptin surge in neonates is the SCAT. Others have reported that *Lep* expression is increased in the WAT of offspring born to dams fed HFD compared to chow during the suckling period [13]; and WAT *Lep* expression correlates with plasma leptin concentration [44] which further supports the primary contribution of WAT to the leptin surge. Since plasma leptin concentrations during the leptin surge are higher in SL than in NL even when adjusted for fat mass, we hypothesize that there could be an enhancer for the *Lep* gene promoter that is only accessible during the nursing period and is regulated by nutritional calorie flux [45, 46].

Using TET-On (dox-dependent) leptin overexpressing mice, we found that dox-induced elevation of leptin during the period of the natural leptin surge (first 3 weeks of life) led to greater weight gain when mice are subsequently exposed to HFD as adults [4]. The dox-augmented leptin surge mimicked the greater leptin surge associated with overnutrition (small litter or maternal HFD) observed in the current study. Consistent with our results, others have reported that IP administration of leptin to nursing pups (P0–10, P10–20, or P3–13) increases weight gain when these animals are offered HFD as adults [47, 48]. Pico et al. reported that oral administration of leptin from P1 to 20 (5 times the mean daily leptin intake from the mother’s milk, increasing doses over the postnatal days from 1 to 44 ng per day) decreased adult body weight [49]. These studies suggest that while circulating leptin likely modulates energy homeostasis through effects on the CNS development, oral leptin may be acting through an independent mechanism mediated directly through gut-brain connections [4, 47–49].

While circulating glucose concentrations during the leptin peak (at P10) were not different among the litters, insulin concentrations were elevated in the SL and correlated with fat mass. In addition to leptin, other metabolic signals—including insulin and ghrelin—have been suggested to act as neurotrophic factors during the postnatal period and to affect subsequent energy homeostasis. Insulin injected into the VMH of P8 rats decreased neuronal density of the VMH by P15 and was associated with increased weight gain in adulthood [50]. Carmody et al. showed that wild-type offspring reared by hyperinsulinemic dams (wild-type pups born to dams heterozygous for a null allele of the insulin receptor), had increased numbers of ARH POMC-expressing cells at P9 and were 14% heavier than offspring of wild-type dams at 4 and 8 weeks of age [51]. Maternal HFD-induced hyperinsulinemia is associated with reduction of the density of the α-MSH fibers in the PVH [12].

Ghrelin has also been implicated in postnatal neurodevelopment [52] and was shown to be transiently elevated on P14 in NL but not in SL mice [18]. In P4 to P22 mice Steculorum et al. found that blocking ghrelin signaling by i.p injections of an anti-ghrelin molecule resulted in increased density of AgRP and α-MSH immunoreactive fibers in the PVH; provision of exogenous ghrelin from P4 to P12 reduced innervation in the PVH [52]. Both interventions resulted in increased body weight, fat, and blood glucose in adult animals. While the overall density of AgRP and αMSH fibers in the PVH were affected in opposite directions by ghrelin and anti-ghrelin treatment, the ratio of orexigenic AgRP to anorexigenic α-MSH terminals was increased in both conditions, possibly accounting for the increased body weight associated with both manipulations [53].

In mice the immediate postnatal period is analogous to the third trimester of gestation in humans [54, 55]. The human-equivalent of the leptin surge might occur in utero. In humans, plasma leptin at birth correlates with gestational age, fetal size, and birth weight, but is not related to maternal circulating leptin concentrations [56]. Leptin concentrations decrease in neonates immediately after birth. Several studies reported up to 13-fold higher leptin concentrations at birth compared to values within a few days of birth [57–60]. In these studies, circulating leptin concentrations after birth correlated with body mass but less leptin was secreted per unit of fat mass compared to during gestation. Leptin concentrations in human fetuses increase over the course of gestation [56, 61–63]. It is possible that in humans the third trimester of gestation is a critical time period during which maternal obesity ‘programs’ future obesity of the offspring. Such programming may be mediated by the elevated leptin concentrations in utero transferred from maternal circulation and/or by the maternal nutritional or metabolic factors that increase leptin production in the fetal adipose tissue. This inference is consistent with data indicating that, in humans, maternal gravid adiposity is positively associated with birth weight [64] and children born large for gestational age are at a higher risk for adult obesity [65].

The strong secular trend to increased prevalence of obesity, occurring earlier in life, clearly reflects environmental changes, some of which might be conveyed by increasing maternal adiposity [66]. Children born to the same mother following bariatric surgery are less frequently obese than siblings born presurgery [67]. Maternal prepregnancy obesity increases the risk of offspring obesity [68], and children with obese fathers are less obese than those with obese mothers [69]. The underlying cellular and molecular mechanisms for maternal programming of adult obesity remain unclear; in rodents, hormonal signals during early postnatal development, including leptin, insulin and ghrelin, mediate these effects—at least in part—via the CNS.

Our data indicate that in mice, nutritional status during the perinatal period is critical in determining the magnitude of the leptin surge, which in turn affects susceptibility to dietary obesity in adulthood. Rodents subjected to early overnutrition become fatter during the suckling period and this increased adiposity persists into adulthood. These offspring are also more susceptible to gain weight when exposed to HFD in adulthood [30, 31]. Our data demonstrate that early overfeeding dramatically augments the postnatal leptin surge, and in the context of studies of isolated postnatal hyperleptinemia [4, 47–49], suggest that susceptibility in adulthood to weight gain when offered a calorically dense palatable diet is mediated—at least in part—by exposure of the developing hypothalamus to high ambient leptin. The mechanism by which leptin is elevated in the postnatal pups under normal conditions and augmented by early overnutrition should be further investigated as it may provide important insights into the developmental programming of adult obesity.

## Supplementary information

Supplementary material
