# Supplementary material for: The postnatal leptin surge in mice is variable in both time and intensity and reflects nutritional status
Source: Int J Obes (Lond). 2021 Sep 2;46(1):39–49. doi: 10.1038/s41366-021-00957-5 (PMC8748198; doi:10.1038/s41366-021-00957-5)
Supplement: Supplementary file 1 — Supplementary material [file 41366_2021_957_MOESM1_ESM.pdf]

The postnatal leptin surge in mice is variable in both time and intensity and reflects nutritional status.

Manuscript Number: 2021IJO00149.

Alicja A. Skowronski[1], Evan D. Shaulson[1], Rudolph L. Leibel[1,2], Charles A. LeDuc[1,2*]

[1]*Division of Molecular Genetics, Department of Pediatrics, Vagelos College of Physicians and Surgeons, Columbia University Irving Medical Center, New York, NY 10032, USA.*

[2]*Naomi Berrie Diabetes Center, Columbia University Irving Medical Center, New York, NY 10032, USA.*

Supplementary Material

Supplementary figure and table legends

Fig. S1. Over- and undernutrition effects on mean plasma leptin concentrations during the first 4 weeks of life in females (A) compared to males (B). (C) AUC for plasma leptin for each sex. Differences in leptin concentration AUC among the groups were tested using two-way ANOVA with Sidak post hoc testing at the 5% level of significance; ns denotes non-significant difference. Means shown with ± SEM.

Fig. S2. Body weight (A), body fat (B) and lean mass (C) in the replication cohort of mixed sexes pups raised in small (SL; 3 pups per dam; orange), normal (NL; 7-8 pups per dam; gray), and large (LL; 11-12 pups per dam; blue) litters that were monitored over the first 4 weeks of life and bled once per week. GEE analysis performed to compare changes in body weight and

composition during the pre-weaning period while pups were in their respective litter size groups; statistics in supplementary Table 3. Different lowercase letters indicate significant difference by the Wald Chi-Square test at the 5% level of significance. Data shown as mean ± SEM.

Fig. S3. Maternal HFD-feeding effects on mean plasma leptin concentrations during the first 4 weeks of life in females (A) compared to males (B). (C) AUC for plasma leptin for each sex. Differences in leptin concentration AUCs among the groups were tested using two-way ANOVA with Sidak post hoc testing at the 5% level of significance; ns denotes non-significant difference. Means shown with ± SEM.

Fig. S4. Over- and undernutrition effects on leptin surge in plasma in the replication cohort bled once per week. (A) Mean plasma leptin concentration, (B) mean leptin concentration AUC over P6-P17, (C) mean leptin concentration AUC over P6-P17 normalized to mean fat mass over that time period, (D) mean peak leptin concentration (peak defined as the maximum leptin concentration measured during the first 4 weeks of postnatal life), (E) mean peak leptin concentration adjusted for body weight and age at which the peak occurred in mixed sexes pups raised in small (SL; 3 pups per dam; orange), normal (NL; 7-8 pups per dam; gray), and large (LL; 11-12 pups per dam; blue) litters that were monitored over the first 4 weeks of life. Differences in leptin AUC and peak leptin concentrations among the groups were tested using one-way ANOVA with Tukey post hoc (or Sidak for ANOVA with covariates) at the 5% level of significance; $*p<0.05$, $**p<0.01$, $***p<0.001$. Means shown with ± SEM.

Fig. S5. Plasma leptin (A) and relative expression of *Lepr* (B), *Adrb3* (C), *Ucp1* (D), *Hsl* (E), and *Adipoq* (F) in SCAT, PGAT, PR, Mes, iBAT, and aBAT in P10 pups reared in small, normal or large litters. Relative expression was normalized to a housekeeping gene, *Actb.* Differences among the groups were tested with one- or two-way ANOVA with Tukey post hoc testing at the 5% level of significance; *p<0.05; **p<0.01, ***p<0.001. Means shown with ± SEM.

Table S1. Sequences for primer-probe mixes used for gene expression assays.

Table S2. Statistical results of the Generalized Estimating Equation (GEE) used to compare body weight, fat mass and lean mass in the small (SL), normal (NL) and large (LL) litters pups of the primary cohort.

Table S3. Statistical results of the Generalized Estimating Equation (GEE) used to compare body weight, fat mass and lean mass in the small (SL), normal (NL) and large (LL) litters pups of the replication cohort.

Table S4. Statistical results of the Generalized Estimating Equation (GEE) used to compare body weight, fat mass and lean mass in the pups raised by dams fed HFD at parturition, or for 20 weeks pre-conception, or control dams fed breeder chow during the same periods.

Fig. S1

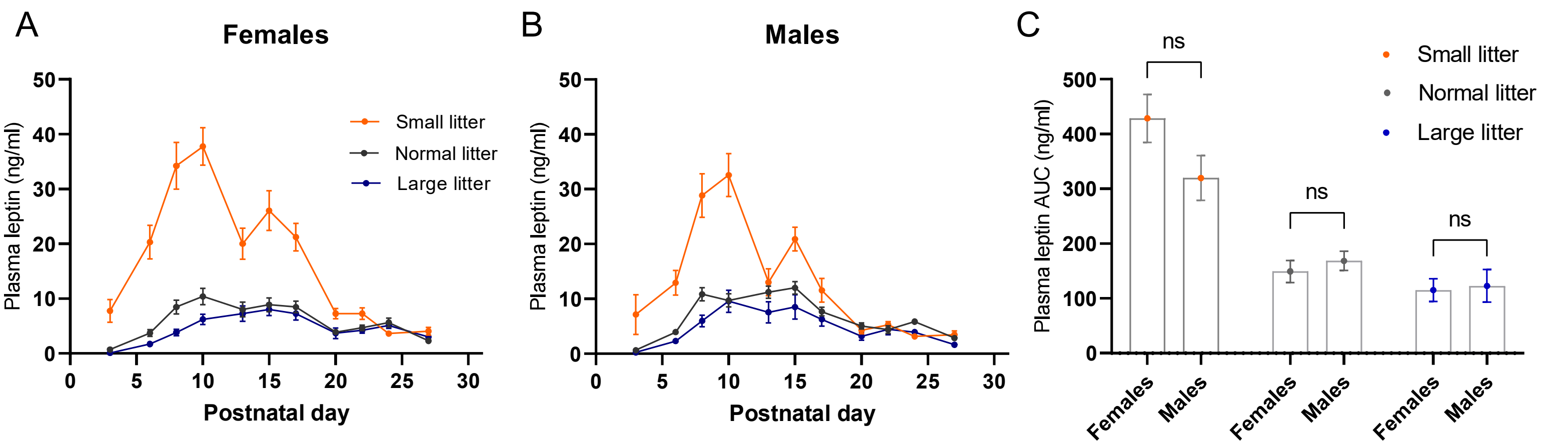
A
Females
Plasma leptin (ng/ml)
Postnatal day
Small litter
Normal litter
Large litter
B
Males
Plasma leptin (ng/ml)
Postnatal day
C
Plasma leptin AUC (ng/ml)
ns
ns
ns
Small litter
Normal litter
Large litter
Females
Males
Females
Males
Females
Males

Fig. S2

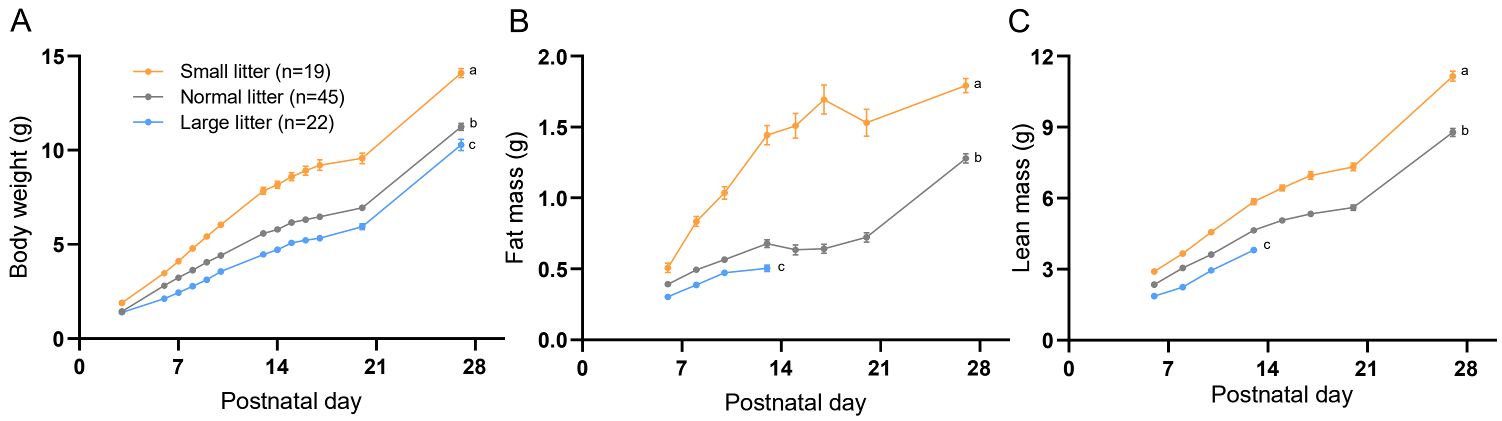
A
B
C
Small litter (n=19)
Normal litter (n=45)
Large litter (n=22)
Body weight (g)
Fat mass (g)
Lean mass (g)
Postnatal day
Postnatal day
Postnatal day
a
b
c
a
b
c
a
b
c

Fig. S3

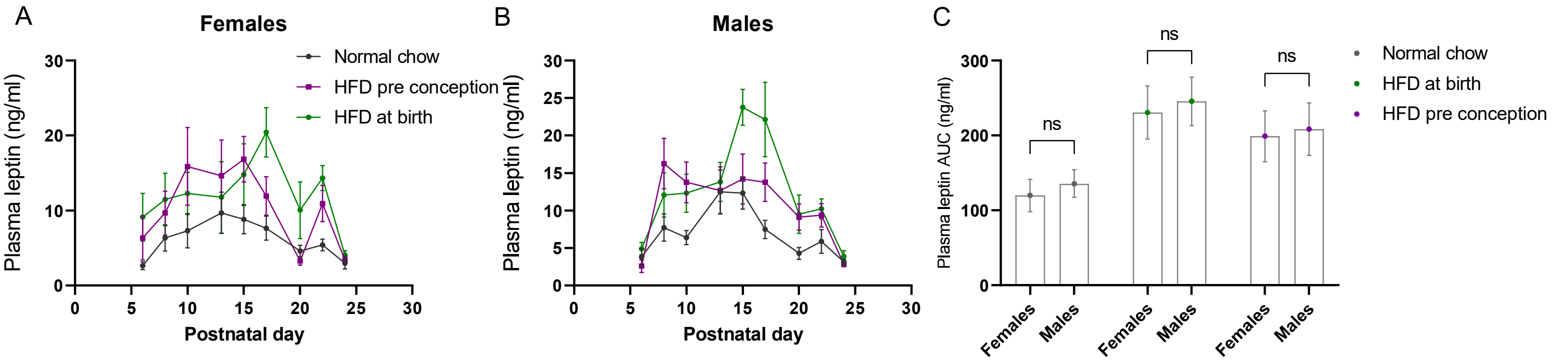
A
Females
Plasma leptin (ng/ml)
Postnatal day
Normal chow
HFD pre conception
HFD at birth
B
Males
C
Plasma leptin AUC (ng/ml)
ns
Females
Males
Normal chow
HFD at birth
HFD pre conception

Fig. S4

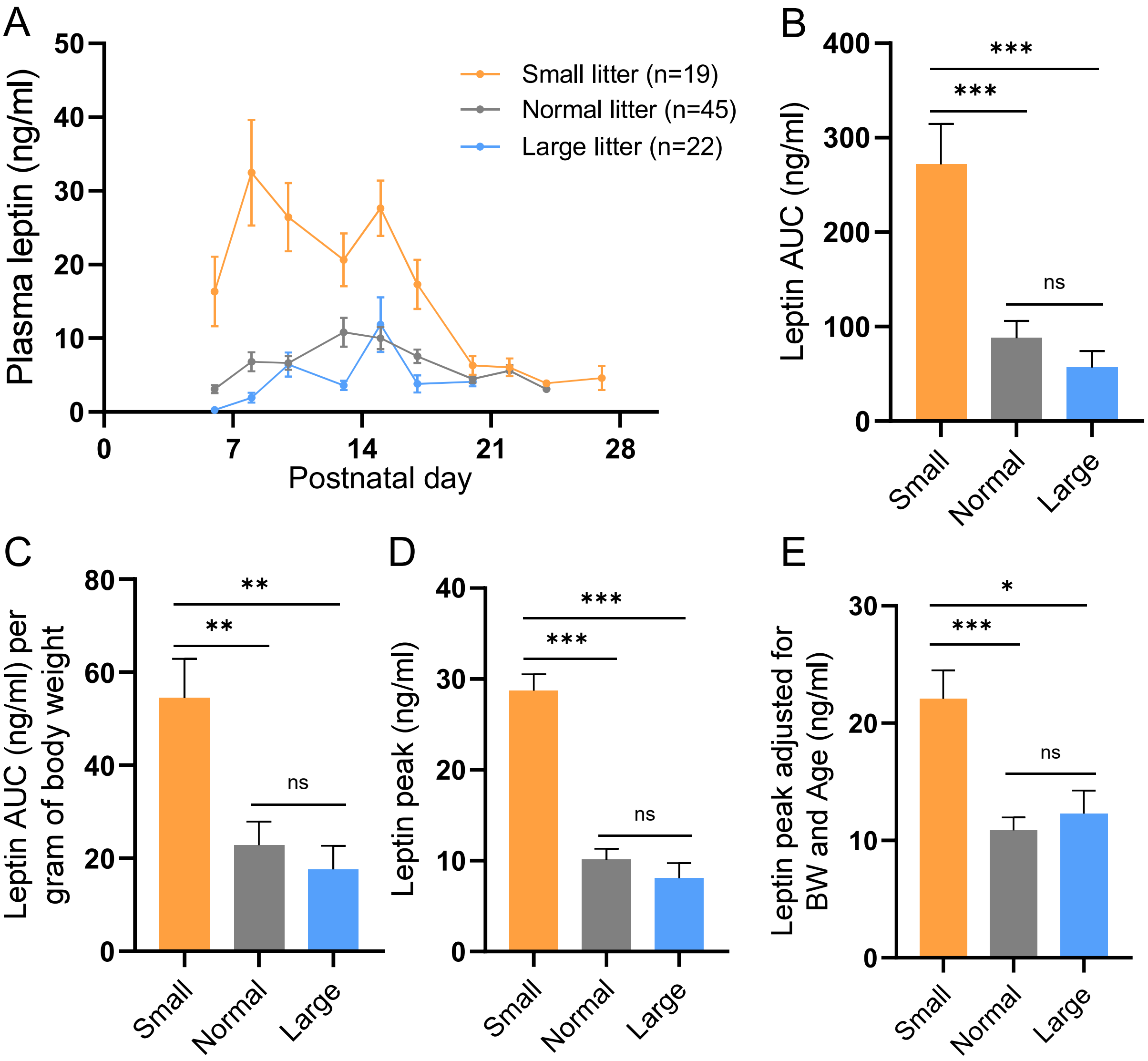
A
Plasma leptin (ng/ml)
Postnatal day
Small litter (n=19)
Normal litter (n=45)
Large litter (n=22)
B
Leptin AUC (ng/ml)
***
***
ns
Small
Normal
Large
C
Leptin AUC (ng/ml) per gram of body weight
**
**
ns
D
Leptin peak (ng/ml)
***
***
ns
E
Leptin peak adjusted for BW and Age (ng/ml)
*
***
ns

Fig. S5

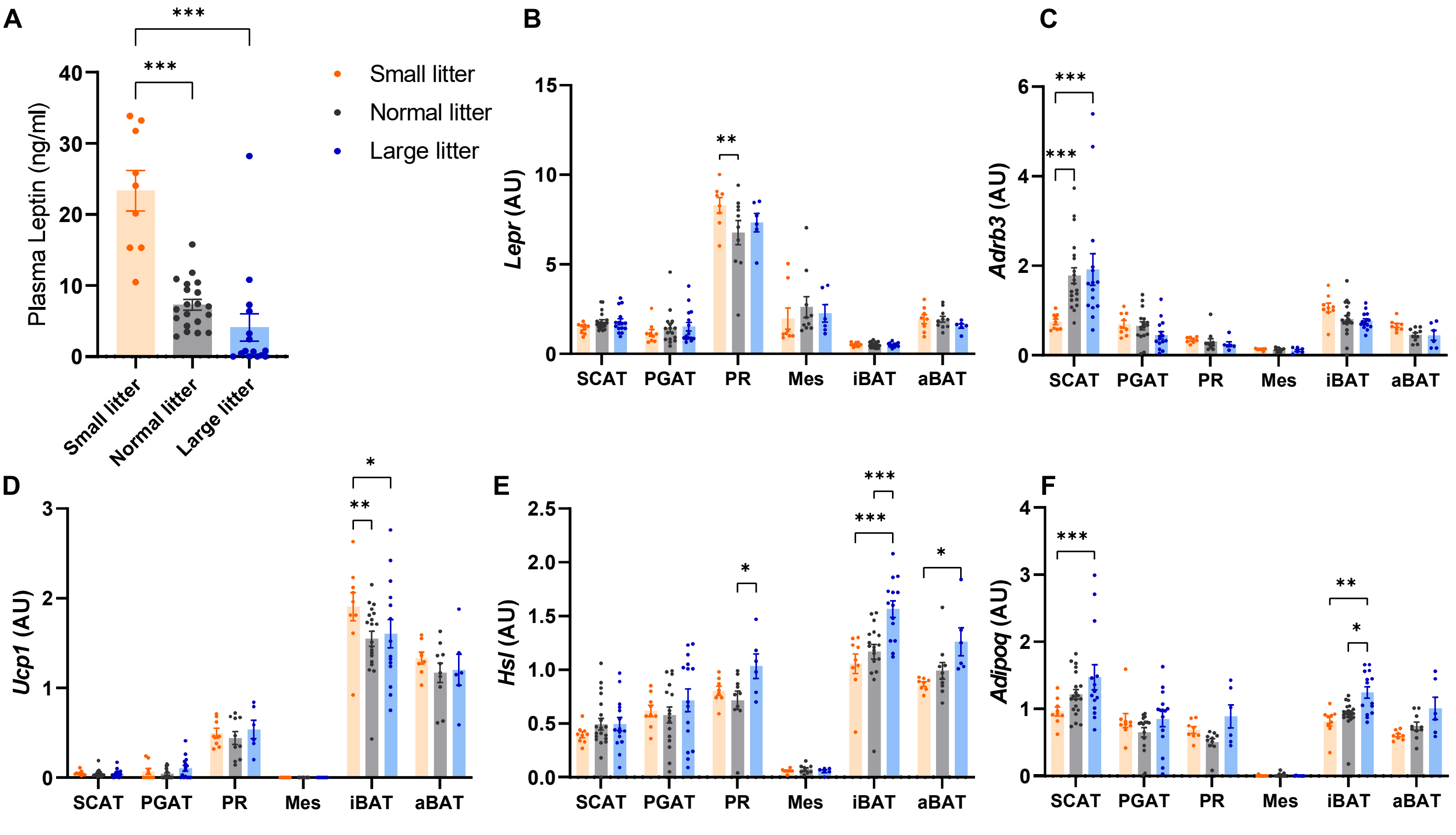
A
B
C
D
E
F
Plasma Leptin (ng/ml)
Small litter
Normal litter
Large litter
Lepr (AU)
Adrb3 (AU)
Ucp1 (AU)
Hsl (AU)
Adipoq (AU)
SCAT
PGAT
PR
Mes
iBAT
aBAT

Supplemental Table 1

Sequences for primer-probe mixes used for gene expression assays

Gene	Sequence	
Actb	Forward	GACTCATCGTACTCCTGCTTG
	Reverse	GATTACTGCTCTGGCTCCTAG
	Probe:	/5HEX/CTGGCCTCA/ZEN/CTGTCCACCTTCC/3IABkFQ/
Lep	Forward	TTCTCCAAGAGCTGCTCC C
	Reverse	CCTGGTGGCCTTTGAAACTT
	Probe:	/5Cy5/AAC AGT TGG /TAO/ATG TTA GCC CTG AAT GCT /3IAbRQSp/
Ptgfr	Forward	GATCTGATTCCACGTTGCCA
	Reverse	GCCATAATGTGCGTCTCCT
	Probe:	/56-FAM/TGGAGTCCC/ZEN/TTTCTGGTAACAATGGC/3IABkFQ/
Ucp1	Forward	CACACCTCCAGTCATTAAGCC
	Reverse	CAAATCAGCTTTGCCTCACTC
	Probe:	/56-FAM/AAACACCTG/ZEN/CCTCTCTCGGAAACAA/3IABkFQ/
Adrb3	Forward	CCAGAAGTCCTGCAAAAACG
	Reverse	CCACCGCTCAACAGGTTT
	Probe:	/5Cy5/CCGTGAAGATCCAGCAAGGAAGCT/3IAbRQSp/
Lepr	Forward	GCTCAGACGTAGGATGAATAGATG
	Reverse	TCACCCAGCACAATCCAAT
	Probe:	/56-FAM/TGAGGTATC/ZEN/ACAGGCGCAGCC/3IABkFQ/
Lipe	Forward	CCATATTGTCTTCTGCGAGTGT
	Reverse	GGCGAAAAGGCAAGATCAAAG
	Probe:	/5Cy5/CGTGCGTAAATCCATGCTGTGTGAGA/3IAbRQSp/
Adipoq	Forward	GCAGGATTAAGAGGAACAGGAG
	Reverse	TGTCTGTACGATTGTCAGTGG
	Probe:	/56-FAM/ACGACACCA/ZEN/AAAGGGCTCAGGAT/3IABkFQ/

Supplemental Table 2

Litter size effects on weight and composition of pups in the primary cohort

Primary Cohort P0-P22		95% Wald Confidence Interval			
Diet Group * Postnatal Day	Beta	Lower	Upper	Wald Chi-Square	Sig
Body weight					
SL vs NL	0.029	0.012	0.046	11.614	*0.001*
LL vs NL	-0.077	-0.091	-0.064	122.086	*<0.001*
LL vs SL	-0.107	-0.121	-0.092	*196.400*	*<0.001*
Fat mass					
SL vs NL	0.01	0.003	0.017	*7.56*	*0.006*
LL vs NL	-0.019	-0.025	-0.013	*36.310*	*<0.001*
LL vs SL	-0.029	-0.035	-0.022	*78.972*	*<0.001*
Lean mass					
SL vs NL	0.052	0.033	0.07	*30.483*	*<0.001*
LL vs NL	-0.035	-0.051	-0.02	*20.136*	*<0.001*
LL vs SL	-0.087	-0.103	-0.071	*111.650*	*<0.001*

Primary Cohort P0-P28		95% Wald Confidence Interval			
Diet Group * Postnatal Day	Beta	Lower	Upper	Wald Chi-Square	Sig
Body weight					
SL vs NL	-0.03	-0.047	-0.012	11.266	*0.001*
LL vs NL	-0.081	-0.095	-0.067	122.857	*<0.001*
LL vs SL	-0.051	-0.067	-0.036	*42.349*	*<0.001*
Fat mass					
SL vs NL	-0.007	-0.013	-0.002	*6.214*	*0.013*
LL vs NL	-0.017	-0.022	-0.012	*42.824*	*<0.001*
LL vs SL	-0.009	-0.014	-0.004	*12.182*	*<0.001*
Lean mass					
SL vs NL	-0.02	-0.038	-0.002	*4.715*	*0.030*
LL vs NL	-0.064	-0.079	-0.048	*64.183*	*<0.001*
LL vs SL	-0.044	-0.06	-0.027	*27.612*	*<0.001*

Supplemental Table 3

Litter size effects on weight and composition of pups in the replication cohort

REPLICATION COHORT		95% Wald Confidence Interval			
Diet Group * Postnatal Day	Beta	Lower	Upper	Wald Chi-Square	Sig
Body weight					
SL vs NL	0.119	0.104	0.134	239.917	*<0.001*
LL vs NL	-0.026	-0.04	-0.011	*12.735*	*<0.001*
LL vs SL	-0.144	-0.161	-0.128	*284.972*	*<0.001*
Fat mass					
SL vs NL	0.014	0.008	0.02	*21.133*	*<0.001*
LL vs NL (analysis from P6 to P13)	-0.011	-0.021	-0.002	*5.263*	*0.022*
LL vs SL (analysis from P6 to P13)	-0.098	-0.112	-0.085	*216.282*	*<0.001*
Lean mass					
SL vs NL	0.094	0.077	0.11	*126.988*	*<0.001*
LL vs NL (analysis from P6 to P13)	-0.038	-0.057	-0.019	*15.441*	*<0.001*
LL vs SL (analysis from P6 to P13)	-0.16	-0.186	-0.134	*148.242*	*<0.001*

Supplemental Table 4

Maternal high fat diet (HFD) effects on weight and composition of pups

Diet Group * Postnatal Day	Beta	95% Wald Confidence Interval Lower	Upper	Wald Chi-Square	Sig
Body weight					
HFD at parturition vs Control chow	0.051	0.04	0.063	75.318	*<0.001*
HFD pre-conception vs Control chow	0.032	0.021	0.043	31.562	*<0.001*
Fat mass					
HFD at parturition vs Control chow	0.022	0.017	0.027	81.632	*<0.001*
HFD pre-conception vs Control chow	0.015	0.011	0.02	44.618	*<0.001*
Lean mass					
HFD at parturition vs Control chow	0.044	0.022	0.067	14.732	*<0.001*
HFD pre-conception vs Control chow	0.029	0.009	0.049	8.144	*0.004*
